# Noninvasive intracranial pressure estimation by orbital subarachnoid space measurement: the Beijing Intracranial and Intraocular Pressure (iCOP) study

**DOI:** 10.1186/cc12841

**Published:** 2013-07-24

**Authors:** Xiaobin Xie, Xiaojun Zhang, Jidi Fu, Huaizhou Wang, Jost B Jonas, Xiaoxia Peng, Guohong Tian, Junfang Xian, Robert Ritch, Lei Li, Zefeng Kang, Shoukang Zhang, Diya Yang, Ningli Wang

**Affiliations:** 1 Eye Hospital of China Academy of Chinese Medical Sciences, Beijing, China; 2 Beijing Tongren Eye Center, Beijing Tongren Hospital, Beijing Ophthalmology and Visual Sciences Key Laboratory, Capital Medical University, 1 Dongjiaominxiang Street, Beijing, Dongcheng District 100730, China; 3 Department of Neurology, Beijing Tongren Hospital, Capital Medical University, Beijing, China; 4 Department of Neurosurgery, Beijing Tongren Hospital, Capital Medical University, Beijing, China; 5 Department of Ophthalmology, Medical Faculty Mannheim of the Ruprecht-Karls-University, Heidelberg, Germany; 6 Department of Epidemiology and Biostatistics, School of Public Health and Family Medicine, Capital Medical University, Beijing, China; 7 Department of Radiology, Beijing Tongren Hospital, Capital Medical University, Beijing, China; 8 Einhorn Clinical Research Center, New York Eye and Ear Infirmary, New York, NY, USA; 9 Department of Ophthalmology, New York Medical College, Valhalla, NY, USA; 10 Beijing Institute of Ophthalmology, Capital Medical University, Beijing Tongren Hospital, 17 Hougou Lane, Chong Wen Men, Beijing 100005, China

## Abstract

**Introduction:**

The orbital subarachnoid space surrounding the optic nerve is continuous with the circulation system for cerebrospinal fluid (CSF) and can be visualized by using magnetic resonance imaging (MRI). We hypothesized that the orbital subarachnoid space width (OSASW) is correlated with and can serve as a surrogate for intracranial pressure (ICP). Our aim was to develop a method for a noninvasive measurement of the intracranial CSF-pressure (CSF-P) based on MRI-assisted OSASW.

**Methods:**

The prospective observational comparative study included neurology patients who underwent lumbar CSF-P measurement and 3.0-Tesla orbital magnetic resonance imaging (MRI) for other clinical reasons. The width of the orbital subarachnoid space (OSASW) around the optic nerve was measured with MRI at 3, 9, and 15 mm behind the globe. The study population was randomly divided into a training group and a test group. After adjusting for body mass index (BMI) and mean arterial blood pressure (MABP), algorithms for the associations between CSF-P and OSASW were calculated in the training group. The algorithms were subsequently verified in the test group. Main outcome measures were the width of the orbital subarachnoid space (OSASW) and the lumbar cerebrospinal fluid pressure (CSF-P).

**Results:**

Seventy-two patients were included in the study. In the training group, the algorithms for the associations between CSF-P and OSASW were as follows: (a) CSF-P = 9.31 × OSASW (at 3 mm) + 0.48 × BMI + 0.14 × MABP-19.94; (b) CSF-P = 16.95 × OSASW (at 9 mm) + 0.39 × BMI + 0.14 × MABP-20.90; and (c) CSF-P = 17.54 × OSASW (at 15 mm) + 0.47 × BMI + 0.13 × MABP-21.52. Applying these algorithms in the independent test group, the measured lumbar CSF-P (13.6 ± 5.1 mm Hg) did not differ significantly from the calculated MRI-derived CSF-P (OSASW at 3 mm: 12.7 ± 4.2 mm Hg (*P* = 0.07); at 9 mm: 13.4 ± 5.1 mm Hg (*P* = 0.35); and at 15 mm: 14.0 ± 4.9 mm Hg (*P* = 0.87)). Intraclass correlation coefficients (ICCs) were higher for the CSF-P assessment based on OSASW at 9 mm and at 15 mm behind the globe (all ICCs, 0.87) than for OSASW measurements at 3 mm (ICC, 0.80).

**Conclusions:**

In patients with normal, moderately decreased or elevated ICP, MRI-assisted measurement of the OSASW appears to be useful for the noninvasive quantitative estimation of ICP, if BMI and MABP as contributing parameters are taken into account.

**Trial registration:**

Clinical trial registered with the Chinese Clinical Trial Registry: ChiCTR-OCC-11001271

## Introduction

Knowledge of intracranial pressure (ICP) is of major importance for the diagnosis of neurologic and neuro-ophthalmologic diseases. The ICP has been measured invasively by lumbar puncture [1]. Noninvasive methods that were explored to estimate the ICP included transcranial Doppler sonography [2], tympanic membrane displacement measurement [3], computed tomography [4], magnetic resonance imaging (MRI) [5], scanning laser tomography of the optic nerve head [6], and venous ophthalmodynamometry [7]. All these techniques, however, had some limitations, such as that transcranial Doppler sonography cannot be used on 10% to 15% of the patients because of the ultrasound not being able to penetrate the skull [8]; venous ophthalmodynamometry could be applied only in patients with elevated ICP without papilledema [9]; or because of the perilymphatic duct being less passable with age, tympanic membrane displacement measurements have a relatively low practicability.

The orbital subarachnoid space around the optic nerve is continuous with the cranial subarachnoid space via the optic nerve canal and can be visualized by using T_2_-weighted MRI with a fat-suppressed sequence [10]. The pressure in the orbital subarachnoid space is correlated with the ICP [11]. Patients with increased ICP have a wider than normal orbital subarachnoid space and vice versa [12-19]. These findings led to the hypothesis that the orbital subarachnoid space width (OSASW) is correlated with, and could serve as a surrogate for, the ICP. Further support for this hypothesis was provided by a study reporting a linear relation between the optic nerve sheath diameter, as measured by sonography, and the lumbar cerebrospinal fluid pressure in 12 patients [20]. In a similar manner, the optic nerve sheath diameter, as measured with MRI, significantly correlated with the ICP in patients with traumatic brain injury [21]. These studies, however, had limitations, such as using sonography with a relative precision for measurements of the diameter of the optic nerve and the OSASW [22], or the studies did not quantitatively assess the ICP [12-21], or the studies addressed only special clinical situations such as acute brain trauma, or the diameter of the optic nerve sheaths as surrogate for the OSASW was measured without taking into account the diameter of the optic nerve.

To avoid these limitations, we conducted this study to test the hypothesis whether the OSASW, as measured by orbital MRI, can be used to estimate the ICP.

## Material and methods

The prospective observational comparative study included patients who consecutively underwent cranial MRI and a lumbar puncture for diagnosis and treatment of neurologic diseases between June 2011 and April 2012. The study protocol was approved by the Medical Ethics Committee of the Beijing Tongren Hospital, according to the Declaration of Helsinki, and all patients signed a written informed consent. The study was registered in the Chinese Clinical Trial Registry (registration site: ChiCTR-OCC-11001271). Exclusion criteria for the study were bilateral optic neuritis, optic nerve tumors, ocular or intracranial tumors, visual acuity worse than 20/400, any orbital disease, any cranial surgery, traumatic brain injury, previous lumbar puncture, which may cause hemorrhage within the CSF circulation system and result in obstruction of the spinal subarachnoid space, and the inability to perform an MRI examination properly.

All patients underwent a complete neurologic and ophthalmologic examination, cranial and orbital MRI, and lumbar CSF-P measurement. Body weight and height were measured. The ophthalmologic examination included visual acuity assessment, refractometry, tonometry, slit lamp-assisted biomicroscopy of the anterior and posterior segment of the eye, ophthalmoscopy, and peripapillary retinal nerve fiber layer thickness measurement with spectral domain optical coherence tomography (RTVue-100; software version 4.0; Optovue, Inc., Fremont, CA, USA).

The MRI of the orbital part of the optic nerve/sheath complex was performed at 14:00 hours in a standardized manner in supine position. We used a 3.0-Tesla whole-body scanner (Signa HDx; General Electric Medical System, Milwaukee, WI, USA) equipped with an eight-channel phased-array head coil. To avoid artifacts due to motion of the eye, all subjects were instructed to fixate on a target attached directly in the gantry of the MRI scanner with the eye in primary gaze. Both eyes of the patients were examined in the same manner. If a motion artifact was detected during the study, the sequence was repeated.

For the measurement of the optic nerve/sheath complex, a fast-recovery fast spin-echo sequence (FRFSE) was applied, as described in detail previously [23]. Scout images in the transverse and oblique sagittal planes were used to ensure optimal head positioning; oblique coronal images were used for quantification. Two basic FRFSE sequences were used:

**Figure 1:**
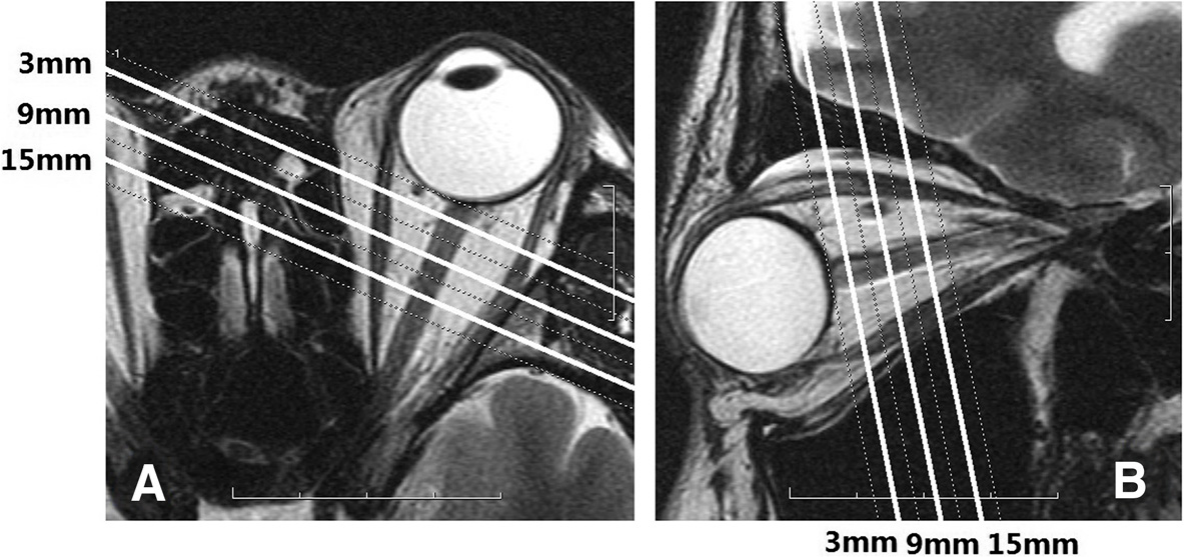
**Magnetic resonance imaging scan of the retrobulbar optic nerve ((A) transversal section, and (B) oblique sagittal section) T2WI-FRFSE of the retrobulbar optic nerve (digital field of view = 8, window width = 2,000, window level = 1,000).** These images were used to plan the position of three slices at 3, 9, and 15 mm behind the globe of T2WI-FRFSE fat-suppressed sequences to measure the optic nerve diameter, optic nerve sheath diameter, and width of the orbital subarachnoid space at 3, 9, and 15 mm behind the globe.

– A T_2_-weighted fast-recovery fast spin-echo sequence (T2WI-FRFSE) that provided good soft-tissue contrast and morphologic data for planning (TR = 2,760 milliseconds; TE = 120 milliseconds; number of excitations = 2; echo train length = 18; bandwidth = 41.67 Hz/pixel; field of view = 16 cm × 16 cm; matrix = 512 × 256; slice thickness = 3 mm; slice gap = 0.3 mm; leading to a nominal spatial resolution of 0.2 mm × 0.2 mm). The sequence was applied twice with 12 contiguous slices in transverse and sagittal orientation. The acquisition time was 1′30″ (transverse) and 1′29″ (sagittal), respectively (Figure 1).

– A T_2_-weighted fast-recovery fast spin-echo sequence with fat suppression was optimized for quantification of the morphology (TR = 6,000 milliseconds; TE = 245 milliseconds; number of excitations = 2; echo-train length = 60; bandwidth = 20.83 Hz/pixel; field of view =16 cm × 16 cm; matrix = 320 × 320; nominal spatial resolution = 0.5 mm × 0.5 mm; slice thickness = 3 mm; slice gap = 0).

**Figure 2:**
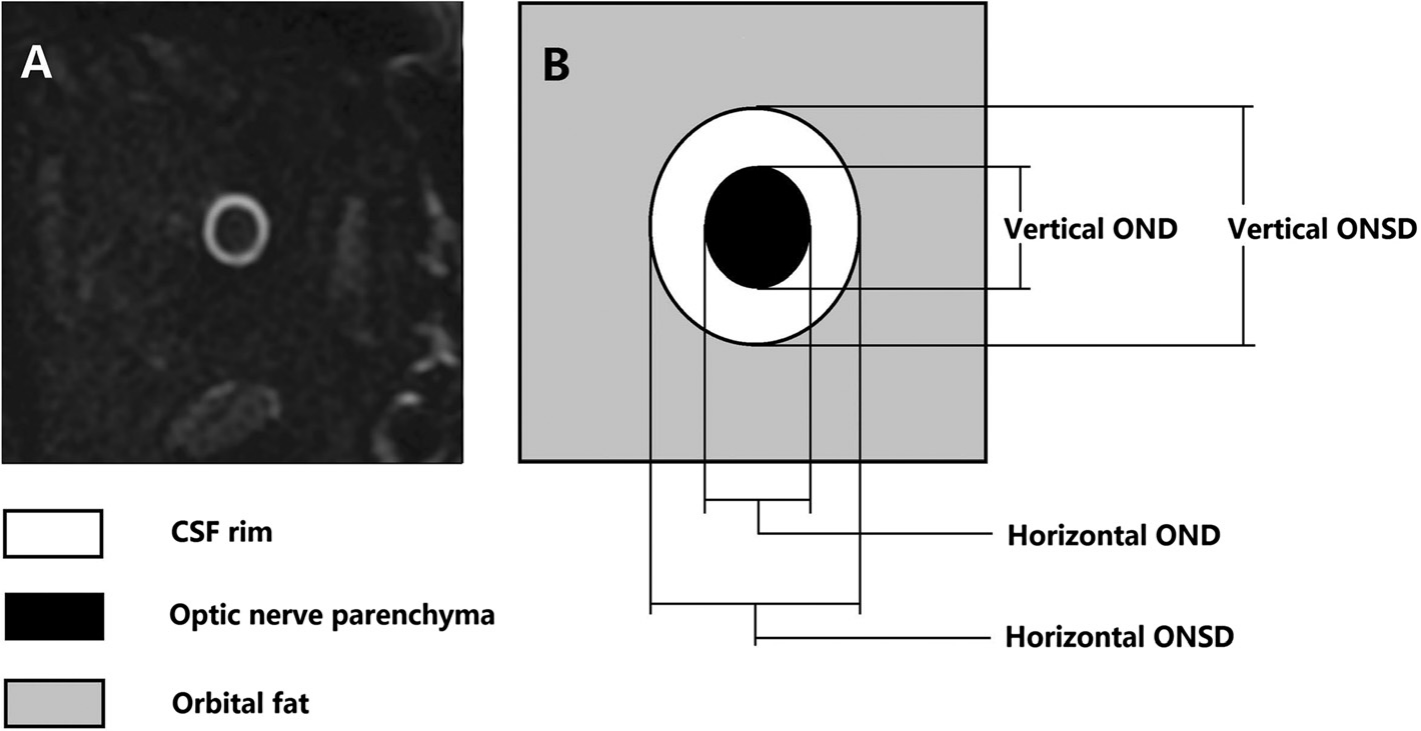
**Scheme to demonstrate the optic nerve/sheath complex. (A)** Oblique coronal T_2_-weighted fast-recovery fast spin-echo sequence (T2WI-FRFSE) image with fat suppression for demonstrating the optic nerve/sheath complex taken at 3 mm behind the globe perpendicular to the optic nerve axis (digital field of view = 4, window width = 2,000, window level = 1,000). The nerve parenchyma is the hypodense area inside of the hypertense ring of cerebrospinal fluid. **(B)** Schematic drawing of the optic nerve/sheath complex including the optic nerve (the black area represents optic nerve), surrounding cerebrospinal fluid space (white rim area), and the optic nerve sheath (at the conjunction of the cerebrospinal fluid space rim and the orbital fat). OND, optic nerve diameter; ONSD, optic nerve sheath diameter.

The T2WI-FRFSE images were interpolated to a higher matrix size of 1,024 × 1,024, leading to a pixel size of 0.16 mm × 0.16 mm for better visualization. Seven oblique coronal MR images were continuously acquired perpendicular to the optic nerve orientation with placement of the first slice directly posterior to the globe. The images were acquired for both eyes separately (Figure 1). The optic-nerve acquisition time was 1′18″. The acquisition time was 11 seconds per slice. In these oblique coronal images, the cerebrospinal fluid (CSF) showed a high, white signal, and the optic nerve parenchyma, a low, dark signal (Figure 2). Three oblique coronal slices perpendicular to the optic nerve at 3, 9, and 15 mm behind the globe were evaluated. Two experienced radiologists (YL, WC) evaluated the images in a masked manner, by using the postprocessing Advantage Workstation 4.4 software (General Electric, Milwaukee, WI, USA).

The horizontal and vertical diameters of the optic nerve and the optic nerve sheath were measured. The average diameter of the optic nerve and of the optic nerve sheaths was calculated as the mean of the measured horizontal and vertical diameters. The width of the optic nerve subarachnoid space was calculated as the difference of half of the optic nerve sheath diameter minus half of the optic nerve diameter (Figure 2) [24]. The measurement results of the first observer were used for the primary analysis. The inter- and intraobserver repeatability was tested on 30 randomly selected individuals from all the patients. For the assessment of the intraobserver repeatability, observer 1 performed the same analysis twice at an interval of 3 months.

The lumbar CSF-P was measured by the same neurologist (GT) in a standardized manner at 14:00 hours in a lateral decubitus position, with the patient’s neck bent in full flexion and the knees bent in full flexion up to the chest. A standard spinal needle (20-gauge, 90 mm in length) was used. The opening pressure was measured. During the procedure, all patients were awake and not sedated. Systolic and diastolic blood pressure was measured in the supine position just before the lumbar puncture was performed. Mean arterial blood pressure was calculated as 1/3 × systolic blood pressure + 2/3 × diastolic blood pressure.

Statistical analysis was performed by using a commercially available statistical software package (SPSS for Windows, version 20.0; IBM-SPSS, Chicago, IL, USA) and the MedCalc program (version 11.5.1.0 for Windows; http://www.medcalc.be; accessed: 2011-4-20). The study population was randomly assigned to a training group and a test group, in a ratio of 4:3. Only one randomly selected unaffected eye per patient was taken for statistical analysis. We determined the mean value (presented as mean ± standard deviation) of the main outcome parameters. The distribution of the values was assessed by using the Kolmogorov-Smirnov test. Differences in the demographic, ophthalmologic, and intracranial characteristics between the training group and the test group were then assessed by using two-tailed Student *t* test. Proportions were compared by using the χ^2^ test. All *P* values were two-sided.

In a first step of the statistical analysis, we used the data of the training group and performed a univariate analysis of the associations between the lumbar CSF-P, MRI-derived orbital measurements, body mass index, mean arterial blood pressure, age, intraocular pressure, and retinal nerve fiber layer thickness.

In a second step, linear, quadratic, and cubic regression models in a multivariate analysis were constructed to assess the associations between lumbar CSF-P and those parameters, which were significantly associated with CSF-P in univariate analysis. Good fitness values and parsimony of the three regression models were compared. The best fit (judged by the r^2^ value) and the most parsimonious one was chosen. Durbin-Watson statistic was used to test for the presence of serial correlations among the residuals [25]. A test for collinearity was performed to test for possible multicollinearity among the independent parameters. A Durbin-Watson statistic between 1.5 and 2.5 indicated that no serious residual autocorrelation was present.

In a third step of the analysis, we tested the value of the calculated mathematical functions to predict the ICP in the test group. A Bland-Altman analysis was applied to measure the prediction’s accuracy and precision [26]. The intraclass correlation coefficient (ICC) and 95% confidence intervals (CIs) of the comparison of both methods were calculated to determine the prediction’s reliability. These procedures were also used to assess the inter- and intraobserver repeatability of the morphologic MRI evaluations.

## Results

**Figure 3:**
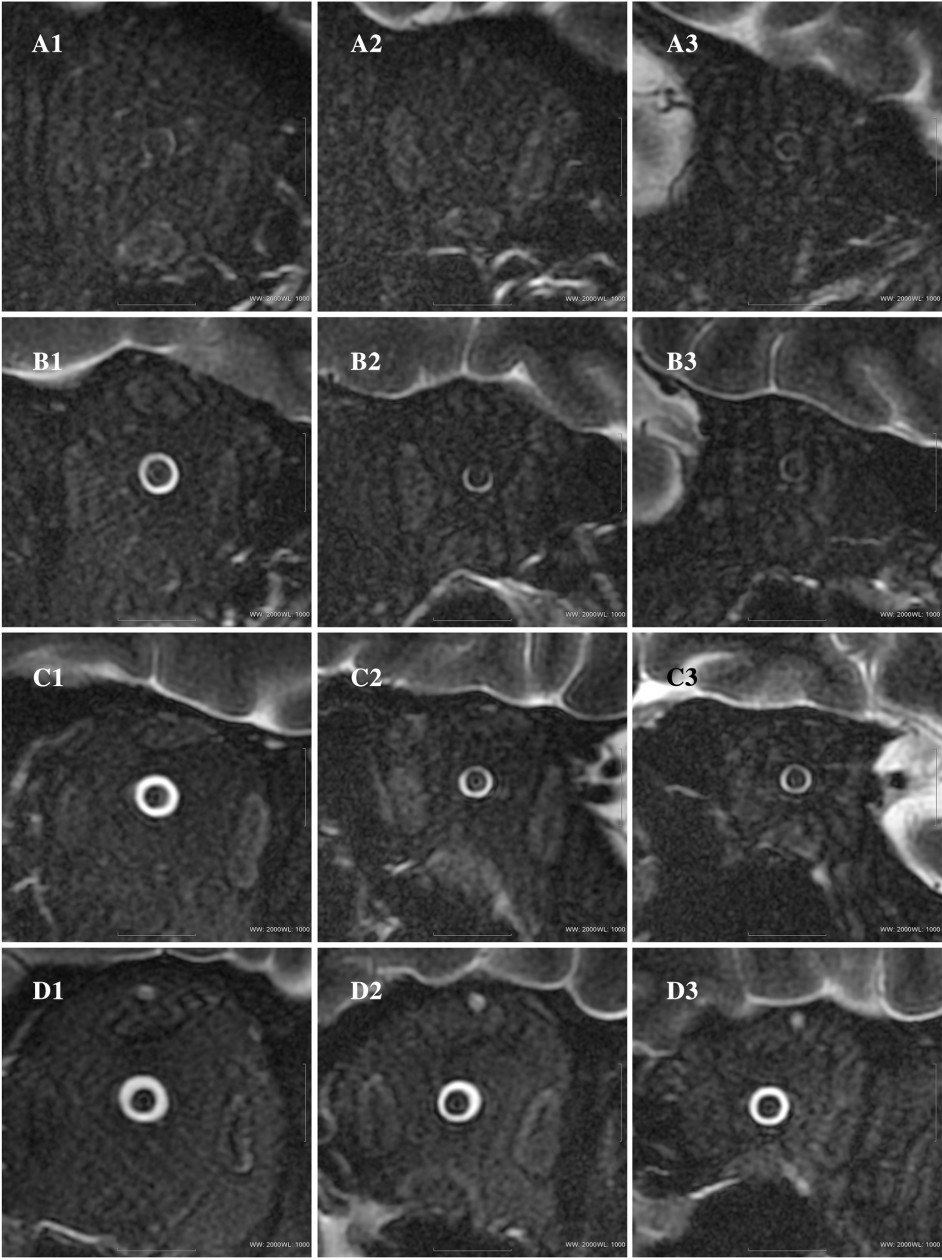
**Oblique magnetic resonance image of the optic nerve/sheath complex (coronal T**_**2**_**-weighted fast-recovery fast spin-echo sequence (T2WI-FRFSE) with fat suppression; digital field of view = 4, window width = 2,000, window level = 1,000), taken at 3 mm (Figure A1, B1, C1, and D1), at 9 mm (Figure A2, B2, C2, and D2), and at 15 mm (Figure A3, B3, C3, and D3) behind the globe.** Figure **A1** to 3: A 70-year-old woman with spontaneous intracranial hypotension; lumbar ICP value was 3.7 mm Hg. Figure **B1** to 3: A 39-year-old man with head injury; lumbar cerebrospinal fluid pressure was 8.5 mm Hg. Figure **C1** to 3: A 33-year-old man with peripheral neuropathy; lumbar cerebrospinal fluid pressure was 16.2 mm Hg. Figure **D1** to 3: A 40-year-old man with idiopathic intracranial hypertension; lumbar cerebrospinal fluid pressure was 24.3 mm Hg.

The study included 72 Han Chinese patients (mean age, 42.0 ± 13.4 years; range, 19 to 70 years), with the data of 42 patients assigned to the training group and the data of the other 30 patients assigned to the test group. The indications for lumbar puncture were peripheral neuropathy, intracranial hypertension, spontaneous intracranial hypotension, cavernous sinus syndrome, meningitis, multiple sclerosis, unilateral ischemic optic neuropathy, unilateral optic neuritis, optic nerve atrophy, and head injury. Because of randomization, the training group and test group did not differ significantly in age, gender, body height and weight, body mass index, intraocular pressure, retinal nerve fiber layer thickness, and arterial blood pressure (all *P* > 0.10). The MRI scans of the OSASW taken at 3 mm behind the globe could be assessed for all patients. Because of image-quality problems, the MRI scans of the OSASW taken at 9 mm behind the globe could not be assessed for three (4.1%) patients, and the MRI scans taken at 15 mm behind the globe could not be assessed for seven (9.5%) patients. Patients with elevated ICP have a wider orbital subarachnoid space than do the patients with decreased ICP (Figure 3).

Including all study participants, the mean optic nerve diameter at 3, 9, and 15 mm behind the globe was 3.16 ± 0.38 mm (media, 3.15 mm; range, 2.30 to 3.95 mm), 2.67 ± 0.43 mm (median, 2.70 mm; range, 1.60 to 3.80 mm), and 2.51 ± 0.46 mm (median, 2.55 mm; range, 1.20 to 3.60 mm), respectively; the optic nerve sheath diameter was 5.09 ± 0.78 mm (median, 5.00 mm; range, 3.60 to 7.65 mm), 4.15 ± 0.70 mm (median, 3.85 mm; range, 2.45 to 5.90 mm), and 3.88 ± 0.70 mm (median, 3.85 mm; range, 2.45 to 5.90 mm), respectively; and the optic nerve sheath width measured 0.96 ± 0.30 mm (median, 0.91 mm; range, 0.52 to 1.95 mm), 0.73 ± 0.20 mm (median, 0.70 mm; range, 0.45 to 1.30 mm), and 0.68 ± 0.18 mm (median, 0.65 mm; range, 0.40 to 1.18 mm), respectively.

**Figure 4:**
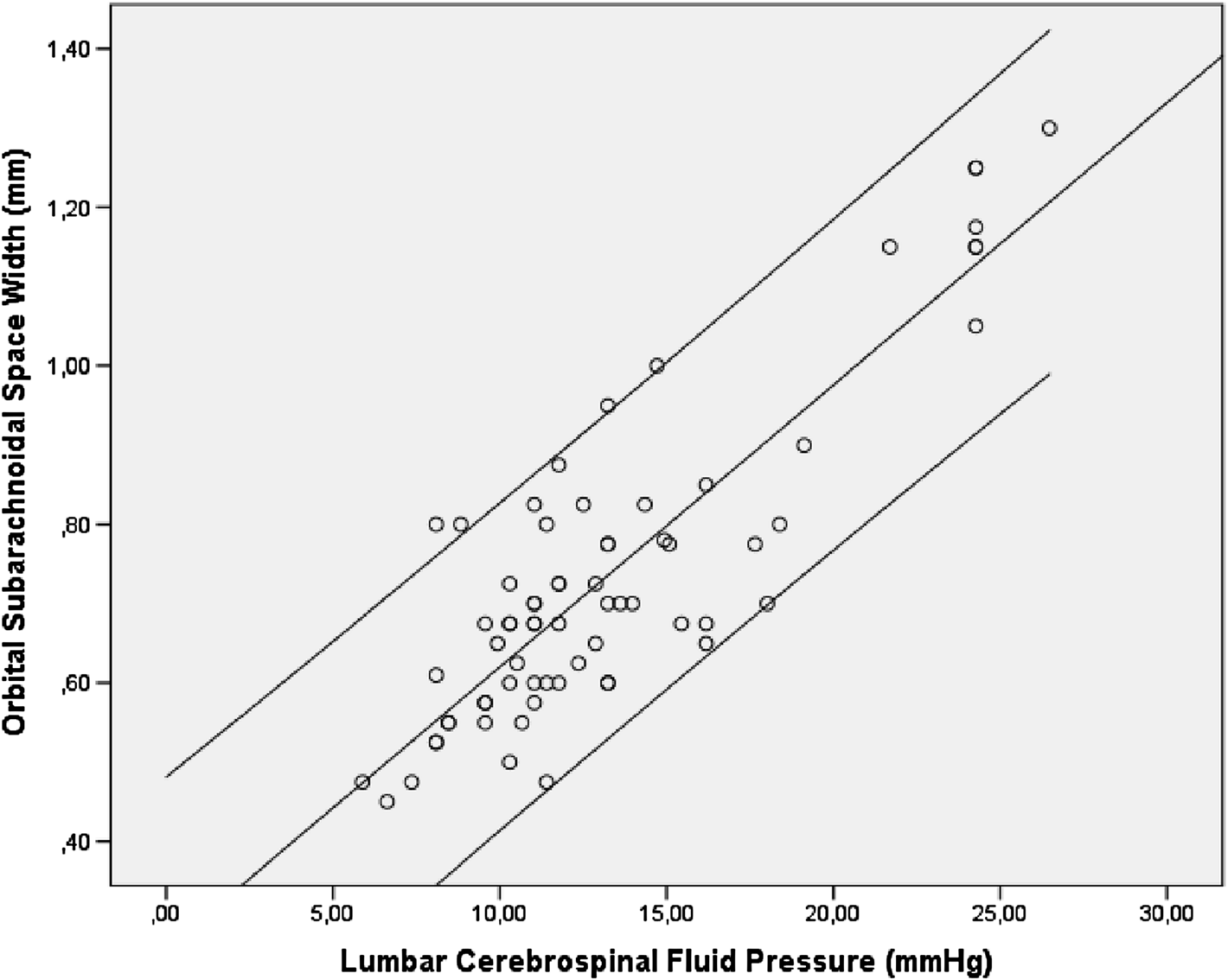
Scattergram showing the distribution of lumbar cerebrospinal fluid pressure measurements versus the width of the orbital subarachnoid space measured at 9 mm behind the globe.

In the training group, lumbar CSF-P was strongly correlated with the OSASW at 3, 9, and 15 mm behind the globe (Pearson correlation *r:* 0.83 ≤ *r* ≤ 0.88; all *P* < 0.0001) (Figure 4). The correlation coefficients for these correlations were higher than those for the associations between lumbar CSF-P and the optic nerve sheath diameters (0.66 ≤ *r* ≤ 0.76; all *P* < 0.0001). The retinal nerve fiber layer thickness was not significantly associated with the OSASW measured at 3, 9, and 15 mm behind the globe (*P* = 0.15; *P* = 0.66; and *P* =0.34, respectively). It was significantly associated with the optic nerve diameter at 9 mm (*r* = 0.61; *P* = 0.001) and 15 mm (*r* = 0.75; *P* < 0.0001) and with the optic nerve sheath diameter at 9 mm (*r* = 0.57; *P* = 0.003) and at 15 mm (*r* = 0.75; *P* < 0.0001). The lumbar CSF-P values were additionally significantly associated with the body mass index (*r* = 0.61; *P* < 0.0001) and mean arterial blood pressure (*r* = 0.55; *P* < 0.0001). In our study population, lumbar CSF-P was not significantly associated with age (*P* = 0.22), intraocular pressure (*P* = 0.67), or retinal nerve fiber-layer thickness (*P* = 0.47).

In the second step of the statistical analysis, different models were tested for the associations between the OSASW and lumbar CSF-P values. The *r*^*2*^ was roughly equal in the linear, quadratic, and cubic models, with the linear model being the most parsimonious. Strong positive linear relations between lumbar CSF-P measurements and the OSASW at 3, 9, and 15 mm were determined within a CSF-P range from 3.7 to 26.5 mm Hg (Table 1). Similarly, lumbar CSF-P showed a moderately positive linear relation with body mass index (BMI) and mean arterial blood pressure (MABP) (Table 1).

**Table 1:** **Comparison of linear, quadratic, and cubic models fit for the association between lumbar CSF-P measurements and OSASWs, BMI, and MABP in the training sample group (*n *** **= 42)**

**Input signal**	**Measurement position**	**Model**	***r***^***2***^	***F***	***P***
OSASW	3 mm behind the globe	Linear	0.70	92.23	<0.0001
		Quadratic	0.71	48.57	<0.0001
		Cubic	0.74	33.22	<0.0001
	9 mm behind the globe	Linear	0.77	126.09	<0.0001
		Quadratic	0.78	65.42	<0.0001
		Cubic	0.80	46.51	<0.0001
	15 mm behind the globe	Linear	0.69	79.93	<0.0001
		Quadratic	0.71	43.59	<0.0001
		Cubic	0.73	30.67	<0.0001
BMI		Linear	0.32	18.93	<0.0001
		Quadratic	0.40	13.21	<0.0001
		Cubic	0.41	13.68	<0.0001
MABP		Linear	0.32	19.19	<0.0001
		Quadratic	0.35	10.60	<0.0001
		Cubic	0.36	10.80	<0.0001

Multivariate analyses were constructed to assess the associations between lumbar CSF-P measurements and orbital subarachnoid space width, lumbar cerebrospinal fluid pressure, and body mass index, which were significantly associated with CSF-P in univariate analysis.

BMI body mass index, Lumbar CSF-P lumbar cerebrospinal fluid pressure, MABP mean arterial blood pressure, OSASW orbital subarachnoid space width.

In the third step, a stepwise multivariate linear-regression analysis revealed that the OSASW (at 3, 9, and 15 mm behind the globe), body mass index, and mean arterial blood pressure were independently associated with lumbar CSF-P measurements. They were entered into multiple regression models (Table 2), out of which three weighting functions for the prediction of ICP derived: 

**Table 2:** **Stepwise multiple linear regression analysis with lumbar CSF-P measurements as the dependent variable in the training group (*n *** **= 42)**

**Regression model**	***r***^***2***^	**Adjusted *r***^***2***^	***F***	***P***^**a**^	**Independent variable**	**Nonstandardized beta**	**Standardized beta**	***t***	***P***^***b***^
Regression model (1) derived from OSASW03	0.83	0.81	59.55	<0.0001	OSASW03	9.31	0.65	8.53	<0.0001
					BMI	0.48	0.27	3.67	0.001
					MABP	0.14	0.22	2.89	0.006
					Constant	−19.94		−4.64	<0.0001
Regression model (2) derived from OSASW09	0.88	0.86	83.92	<0.0001	OSASW 09	16.95	0.70	10.56	<0.0001
					BMI	0.39	0.23	3.54	0.001
					MABP	0.14	0.23	3.43	0.002
					Constant	−20.90		−5.87	<0.0001
Regression model (3) derived from OSASW15	0.81	0.79	47.40	<0.0001	OSASW15	17.54	0.65	7.64	<0.0001
					BMI	0.47	0.27	3.27	0.002
					MABP	0.13	0.21	2.42	0.021
					Constant	−21.52		−4.54	<0.0001

A stepwise multiple linear-regression procedure was conducted to develop mathematical functions of noninvasive ICP assessment, lumbar cerebrospinal fluid pressure measurements as a dependent variable, orbital subarachnoid space width at 3 to 15 mm behind the globe, body mass index, and mean arterial blood pressure as independent variable at the same time. BMI body mass index, lumbar CSF-P lumbar cerebrospinal fluid pressure, MABP mean arterial blood pressure, OSASW03-15, orbital subarachnoid space width at 3 to 15 mm behind the globe.

*P*^a^ for regression models, and *P*^b^ for independent variables.

(1) $$\begin{array}{rl} \mathrm{Non}‒\mathrm{invasive}\;\mathrm{ICP}=\;9 & .31\;\times \;\mathrm{OSASW}03\;+\;0.48\\ & \times \;\mathrm{BMI}\;+\;0.14\;\times \;\mathrm{MABP}\\ & \;–\;19.94 \end{array}$$

(2) $$\begin{array}{rl} \mathrm{Non}‒\mathrm{invasive}\;\mathrm{ICP}= & 16.95\;\times \;\mathrm{OSASW}09\;+\;0.39\\ & \;\times \;\mathrm{BMI}\;+\;0.14\;\times \;\mathrm{MABP}\\ & \;–\;20.90 \end{array}$$

(3) $$\begin{array}{rl} \mathrm{Non}‒\mathrm{invasive}\;\mathrm{ICP}\;=\;17 & .54\;\times \;\mathrm{OSASW}15\;+\;0.47\\ & \times \;\mathrm{BMI}\;+\;0.13\;\times \;\mathrm{MABP}\\ & \;–\;21.52 \end{array}$$

OSASW03, OSASW09 and OSASW15: orbital subarachnoid space width at 3, 9, and 15 mm behind the globe

BMI, body mass index;

ICP, intracranial cerebral fluid pressure;

MABP, mean arterial blood pressure.

The Durbin-Watson value of function (1), (2), and (3) was 2.43, 1.66, and 1.61, respectively. Values falling into the acceptable range of 1.5 to 2.5 indicated a nonsignificant autocorrelation for the residuals in the multiple regression models. The condition index of function (1), (2), and (3) was 35.19, 34.64, and 34.43, respectively.

**Figure 5:**
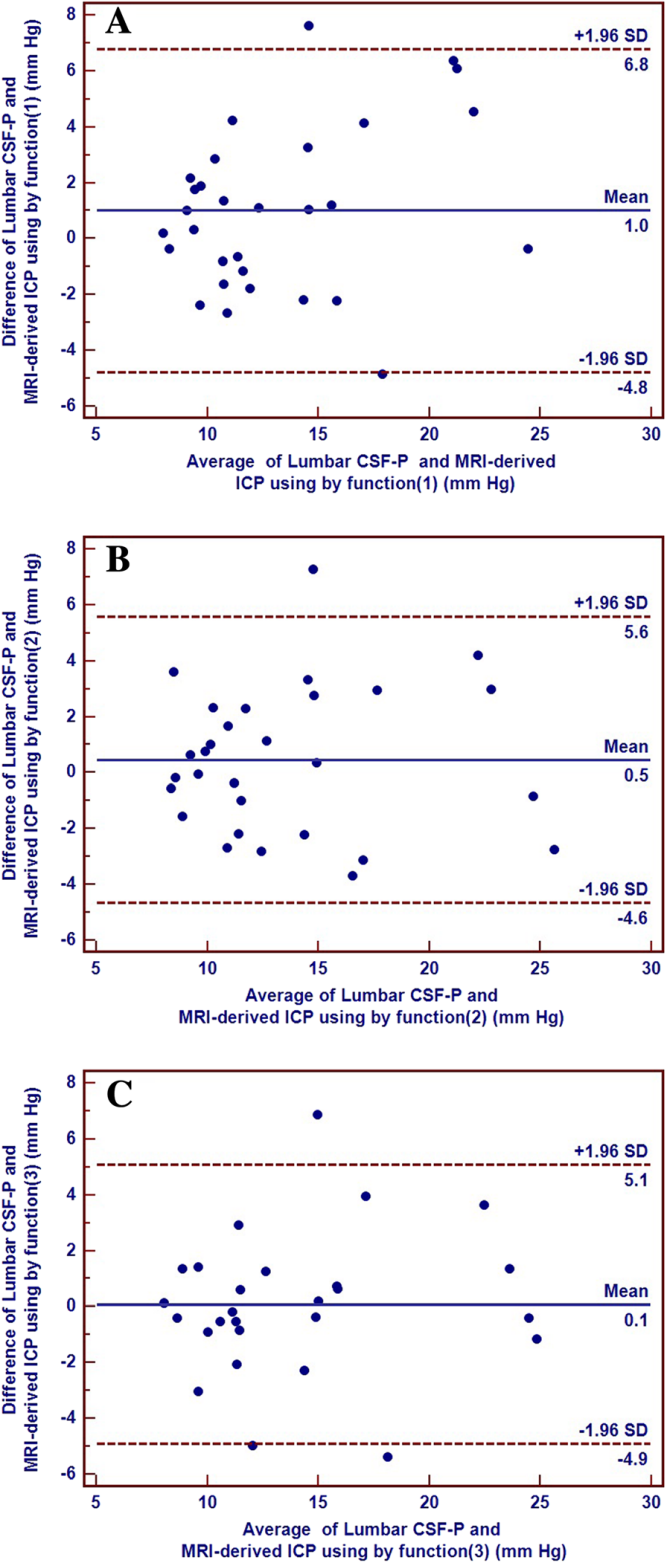
**Bland-Altman graph of the inter-method agreement of the non-invasive magnetic resonance imaging (MRI) derived intracranial pressure (ICP) assessment using weighting function (1) (A), (2) (B), and (3) (C) and invasive lumbar cerebrospinal fluid pressure (CSF-P) measurements in the test group.** CSF-P, cerebrospinal fluid pressure; ICP, intracranial pressure; SD, standard deviation. The solid line represents the mean of the difference between both methods; the two dashed lines represent the mean of the difference plus or minus the 1.96-fold standard deviation of the difference.

In the fourth step of the statistical analysis, the three algorithms derived earlier were applied in the test group. It showed that the measured lumbar CSF-P (13.6 ± 5.1 mm Hg) did not differ significantly from the calculated MRI-derived mean ICP values obtained by using the three weighting functions (weighting function 1, 12.7 ± 4.2 mm Hg (*P* = 0.07); weighting function 2, 13.4 ± 5.1 mm Hg (*P* = 0.35); and weighting function 3, 14.0 ± 4.9 mm Hg (*P* = 0.87). The mean estimated bias ± standard deviation and the 95% limits of agreement suggested that greater accuracy and precision was achieved from functions (2) and (3) than from function (1). The results of the Bland-Altman analysis are shown in Figure 5. All intraclass correlation coefficients (ICCs) of the two methods reached values ≥0.80. The ICCs and their 95% CIs for the noninvasive ICP assessment by using function (1), (2), and (3) were 0.80 (0.62 to 0.90), 0.87 (0.74 to 0.94), and 0.87 (0.74 to 0.94), respectively, which suggested that the reliability of the noninvasive ICP assessment using functions (2) and (3) was higher than the function (1).

The data on the intraobserver and interobserver reproducibility of the optic nerve-sheath complex measurements by MRI, including the 95% limits of agreement of the mean differences, suggested that the intraobserver agreement was superior to the interobserver agreement (Table 3). For both, intraobserver measurements and interobserver measurements, the ICCs, and their 95% CIs were better for the measurements taken closer to the globe.

**Table 3:** Interobserver and intraobserver repeatability of the optic nerve-sheath complex measurements by MRI

**Measurement**	**Interobserver**^**a**^		**Intraobserver**^**b**^	
	**Difference (95% LoA) (mm)**	**ICC (95% CI)**	**Difference (95% LoA) (mm)**	**ICC (95% CI)**
Optic nerve-sheath diameter				
At 3 mm behind the globe	0.17 (−0.10-0.45)	0.96 (0.93–0.98)	−0.04 (−0.43-0.35)	0.99 (0.99–0.99)
At 9 mm behind the globe	0.23 (−0.31-0.77)	0.95 (0.90–0.98)	−0.01 (−0.38-0.36)	0.96 (0.92–0.98)
At 15 mm behind the globe	0.22 (−0.10-0.54)	0.97 (0.93–0.98)	−0.08 (−0.37-0.20)	0.98 (0.97–0.99)
Optic nerve diameter				
At 3 mm behind the globe	0.17 (−0.10-0.40)	0.92 (0.84–0.96)	−0.15 (−0.42-0.12)	0.99 (0.99–0.99)
At 9 mm behind the globe	0.20 (−0.11-0.51)	0.86 (0.74–0.93)	−0.18 (−0.46-0.11)	0.94 (0.88–0.97)
At 15 mm behind the globe	0.21 (−0.12-0.54)	0.84 (0.68–0.92)	−0.17 (−0.40-0.10)	0.97 (0.95–0.98)

The intraclass correlation coefficient (ICC) was calculated to determine the degree of interobserver and intraobserver reliability. The intraobserver and interobserver variabilities were determined by Bland–Altman analysis. *CI* confidence interval, *LoAs* limits of agreement. ^a^Based on the first measurements of the first observer and measurements of the second observer. ^b^Based on the first and second measurements of the first observer.

## Discussion

Within the range of a lumbar CSF-P between 3.7 mm Hg and 26.5 mm Hg, the ICP determined by lumbar puncture was significantly correlated with the OSASW, as determined with MRI. Good fitness was roughly equal in the linear, quadratic, and cubic models of the relation between MRI-determined OSASW and lumbar CSF-P. Because the linear model was the most parsimonious, we chose the linear-fitting equation for further analysis. After adjusting for body mass index and mean arterial blood pressure, two formulas were formed to calculate the ICP, based on the MRI measurements of the OSASW at 9 mm or at 15 mm behind the globe. Applied in an independent test group, these formulas were relatively precise in predicting the ICP. The 3.0-T MRI protocols, based on T2WI-FRFSE with fat-suppression sequences, depicted the orbital optic nerve-sheath complex in its full length with a pixel resolution of 0.16 × 0.16 mm. At the same time, the image-acquisition time (11 seconds per slice) was decreased, thus reducing the risk of potential motion artifacts.

For the control, we assessed the interobserver and intraobserver reproducibility and variability to ascertain the quality of the image analyses. In our evaluation, the relatively high ICC (≥0.84) and low difference (≤0.23 mm) suggested that the standardized region-of-interest evaluation was sufficiently reliable and reproducible.

Our study confirmed previous investigations [12-21]. The anatomic basis for our results was the observation of free communication of CSF between the intracranial cavity and the orbital space through the optic nerve canal [10]. The physiological explanation for our results was that the pressure in the orbital subarachnoid space is correlated with the ICP, and that the orbital subarachnoid space can distend, depending on its pressure, because of the principles of elasticity, according to the Poisson effect. Correspondingly, patients with elevated ICP had a wide orbital CSF space, whereas patients with intracranial hypotension showed a shallow orbital CSF space [12-18]. A linear relation between invasive ICP measurements and the optic nerve-sheath diameter was reported in previous studies on patients with traumatic brain injury [20,21]. In these studies, the optic nerve-sheath diameter showed lower correlation coefficients (0.66 ≤ *r* ≤ 0.76) for the associations with lumbar CSF-pressure measurements than did the OSASW in our study (0.83 ≤ *r* ≤ 0.88). Correspondingly, the retinal nerve fiber-layer thickness as a surrogate for the status of the optic nerve was strongly related to the optic nerve diameter (*r* = 0.61; *P* < 0.0001 at 9 mm; and *r* = 0.75; *P* < 0.0001 at 15 mm behind the globe), and the optic nerve-sheath diameter (*r* = 0.57, *P* = 0.0001 at 9 mm; *r* = 0.75, *P* < 0.0001 at 15 mm) in our study, whereas it was not related to the OSASW. It showed that the OSASW, as compared with the optic nerve-sheath diameter, was a better parameter to assess the ICP.

Our study confirmed previous investigations on the association of lumbar CSF-P measurements with body mass index and with arterial blood pressure [27-29]. It extends these findings to correlations between arterial blood pressure and body mass index and the OSASW.

The results of our study may have clinical implications. In patients with a presumed abnormal ICP, an MRI examination of the orbit with determination of the OSASW may be helpful to derive a hint for the ICP, provided that free communication exists between the intracranial CSF space and the orbital CSF space. From a practical point of view, the technique described in our study might be of particular interest, if a patient in a medical-emergency situation underwent a cranial MRI examination including the orbit for other reasons, so that the same image taken for information about the brain or head can simultaneously serve to find an estimation of the ICP. This may also be important for an early diagnosis of an elevated ICP, because one may assume that, in patients with acutely elevated ICP, the orbital subarachnoidal space may dilate earlier than papilledema of the optic nerve head develops.

Another clinical impact of the technique described in our study may be for patients with a chronic disease. Recent studies suggested that patients with normal- (intraocular) pressure glaucoma may have a low ICP and thus a narrow OSASW [23,30,31]. As a corollary, the increased ICP in patients with idiopathic intracranial hypertension may be estimated by an orbital MRI examination showing a dilated orbital CSF space. Applying the technique described in our study may thus be diagnostically helpful also for patients with a chronic disease associated with an abnormal ICP.

Although our study included 72 patients, only eight of them had an ICP >20 mm Hg. It may suggest that the results can primarily be applied only to patients without increased ICP and that the results give a hint of the ICP estimation in patients with markedly elevated ICP. Moreover, it should be noted that all CSF-P values in the present study were less than 26.5 mm Hg. Because patients with marked high ICP were not included and because the elasticity module of the orbital optic nerve meninges have not been tested, the possibility exists that the relation between CSF-P and OSASW is not linear but shows a plateau in the case of a higher ICP, particularly more than 30 mm Hg.

However, Hansen and colleagues [32] investigated the acute pressure-dependent behavior of the optic nerve-sheath diameter *in vitro* after controlled application of incremental pressure steps in the OSASW. They found that the relation of step magnitude and corresponding ONSD changes was nearly linear within a wide range of 5 to 65 mm Hg (*r* = 0.94; *P* < 0.01) [32]. Even so, when lumbar CSF-P is more than 30 mm Hg, the exact pressure-OSASW relation should be tested in future clinical study.

Potential limitations of our study should be mentioned. First, the MRI examination and the lumbar puncture were not performed at the same time. The MRI examination was carried out in the supine position 24 to 48 hours before the lumbar puncture, which was performed in the left lateral decubitus position. Because both parameters, the OSASW and the lumbar CSF-pressure, show interday variations, the correlations between OSASW and lumbar CSF-pressure measurements might have been higher had both examinations been performed at the same time.

Second, besides OSASW, body mass index, and arterial blood pressure, other factors may be associated with ICP and may have to be incorporated into the formulas for the calculation of ICP.

Third, the study included Han Chinese, and other ethnic groups could differ in the weighting functions to predict the ICP. Therefore, it remains to be determined whether our study results can be applied to patients of other ethnicity.

Fourth, three-dimensional volumetric measurements of the orbital subarachnoid space compared with the two-dimensional measurements as taken in our study may have resulted in higher correlations with the lumbar CSF-P measurements.

Fifth, the method depends on the pathway from the intracranial to the orbital portion of the subarachnoid space of the optic nerve. If that pathway in the optic nerve canal or at the inner aperture of the optic nerve canal is blocked (for example, by a suprasellar meningioma, by circular adhesions as a sequel of a tuberculous meningitis, in patients with obstructive hydrocephalus, or in patients with an intracanalicular ophthalmic artery aneurysm), the orbital CSF-P (and thus the width of the orbital CSF space) is not related to the ICP [33,34].

Sixth, it should be stressed that, based on the MRI calculations, the CSF-P as measured by lumbar puncture could vary by a factor of 2, so that it remains unclear whether the MRI calculations may or may not supplant lumbar-puncture measurements in the evaluation of patients with suspected abnormalities of the CSF-P. The MRI technique may be of value in choosing patients who may need invasive monitoring and the follow-up of such patients.

Future studies may address the question by which means this variation can be further reduced, and with which technique the lumbar-puncture method of the MRI measurement of the orbital CSF space may be more valid to assess the CSF-P.

## Conclusions

In patients with normal, moderately decreased, or elevated intracranial pressure, the latter could noninvasively be estimated based on MRI-assisted OSASW measurements with adjustment for body mass index and mean arterial blood pressure. If confirmed and further refined by future investigations, this finding could open the possibility to measure the intracranial pressure noninvasively. Although this technique will not replace invasive ICP monitoring, it might be helpful and a supplement in choosing patients who may need an invasive examination.

## Key message

• The intracranial pressure can be estimated noninvasively, based on measurements of the width of the orbital cerebrospinal fluid space.

## Abbreviations

BMI: Body mass index; CI: confidence interval; CSF: cerebrospinal fluid; CSF-P: intracranial CSF pressure; FRFSE: fast-recovery fast spin-echo sequence; ICC: intraclass correlation coefficient; ICP: intracranial pressure; MABP: mean arterial blood pressure; MRI: magnetic resonance imaging; OSASW: orbital subarachnoid space width; T2WI-FRFSE: T_2_-weighted fast-recovery fast spin-echo sequence.

## Competing interests

The authors declare that they have no competing interests.

## Authors’ contributions

XBX, JDF, XJZ, HW, JBJ, XXP, GHT, JX, RR, LL, ZFK, SKZ, DY, and NW designed the study. XBX, JDF, XJZ, HW, XXP, GHT, JX, RR, LL, ZFK, SKZ, DY, and NW performed the examinations. XBX, JBJ, XXP, LL, and NW carried out the statistical analysis. XBX, JBJ, RR, and NW drafted the manuscript. All authors read, revised and approved the final manuscript.

## Members of the Beijing iCOP study group

Xiaobin Xie, MD^1,2^, Jost B. Jonas, MD^3^, Junfang Xian MD^4^, Diya Yang PhD^2^, Xiaojun Zhang MD^5^, Guohong Tian MD^5^, Jidi Fu MD^6^, Ruowu Hou MD^6^, Zhen Li MD^7^, Zheng Zhang MD^2^, Jinghong Sang MD^2^, Weiwei Chen PhD^2^, Sumeng Liu MD^2^, and Ningli Wang MD^2,8^.

^1^Eye Hospital of China Academy of Chinese Medical Sciences, Beijing, China.

^2^Beijing Tongren Eye Center, Beijing Tongren Hospital, Capital Medical University, Beijing Ophthalmology and Visual Sciences Key Laboratory, Beijing, China.

^3^Department of Ophthalmology, Medical Faculty Mannheim of the Ruprecht-Karls-University Heidelberg, Germany.;

^4^Department of Radiology, Beijing Tongren Hospital, Capital Medical University, Beijing, China.

^5^Department of Neurology, Beijing Tongren Hospital, Capital Medical University, Beijing, China.

^6^Department of Neurosurgery, Beijing Tongren Hospital, Capital Medical University, Beijing, China.

^7^Department of Ophthalmology, Xuan Wu Hospital, Capital Medical University, Beijing China.

^8^Beijing Institute of Ophthalmology, Beijing Tongren Hospital, Capital Medical University, Beijing, China.
